# Supplementary material for: BHLHE40/41 regulate macrophage/microglia responses associated with Alzheimer’s disease and other disorders of lipid-rich tissues
Source: bioRxiv. 2023 Feb 13:2023.02.13.528372. Preprint. [Version 1] doi: 10.1101/2023.02.13.528372 (PMC9948946; doi:10.1101/2023.02.13.528372)
Supplement: Supplement 7 [file NIHPP2023.02.13.528372v1-supplement-7.pdf]

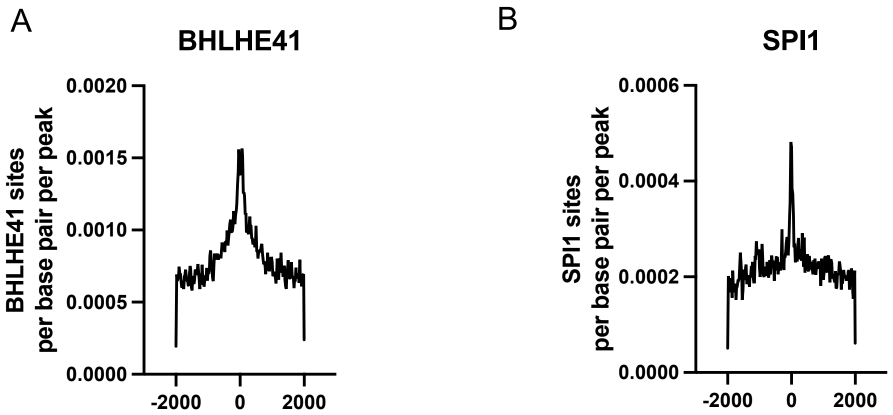

Supplementary Figure 1. BHLHE41 and SPI1/PU.1 regulons are most highly enriched in direct targets. Frequency of **A)** BHLHE41 and **B)** SPI1/PU.1 motifs are plotted against the distance from the microglial ATAC-seq peaks[31] in the promoters of genes in their regulons.

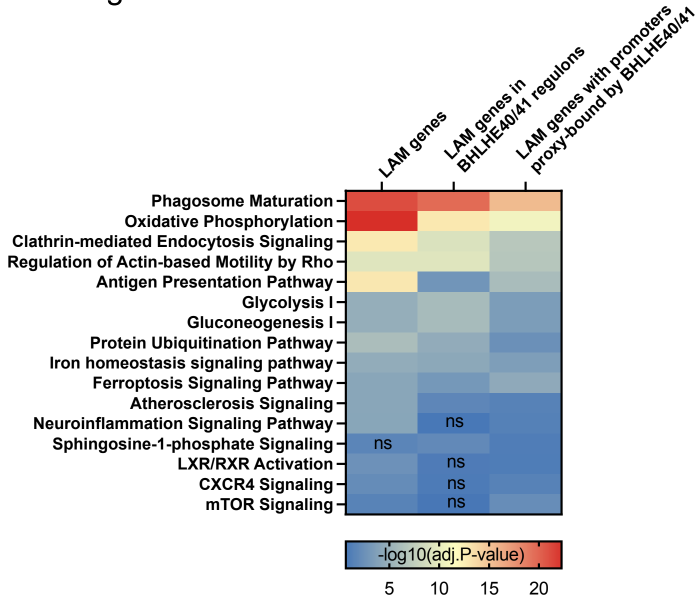

Supplementary Figure 2. Pathways shared between lipid-associated macrophage (LAM) genes with promoter proxy-bound by BHLHE40/41 and LAM genes in BHLHE40/41 regulons. Canonical pathways (IPA) shared between human LAM genes (Jaitin *et al.* [3], Dataset S6, FDR Adj.P-value < 0.05), LAM genes proxy-bound by BHLHE40/41, and LAM genes from BHLHE40/41 regulons. Adj.P-value represents Bonferroni-Holm correction. ns – non significant (Adj.P-value > 0.05).

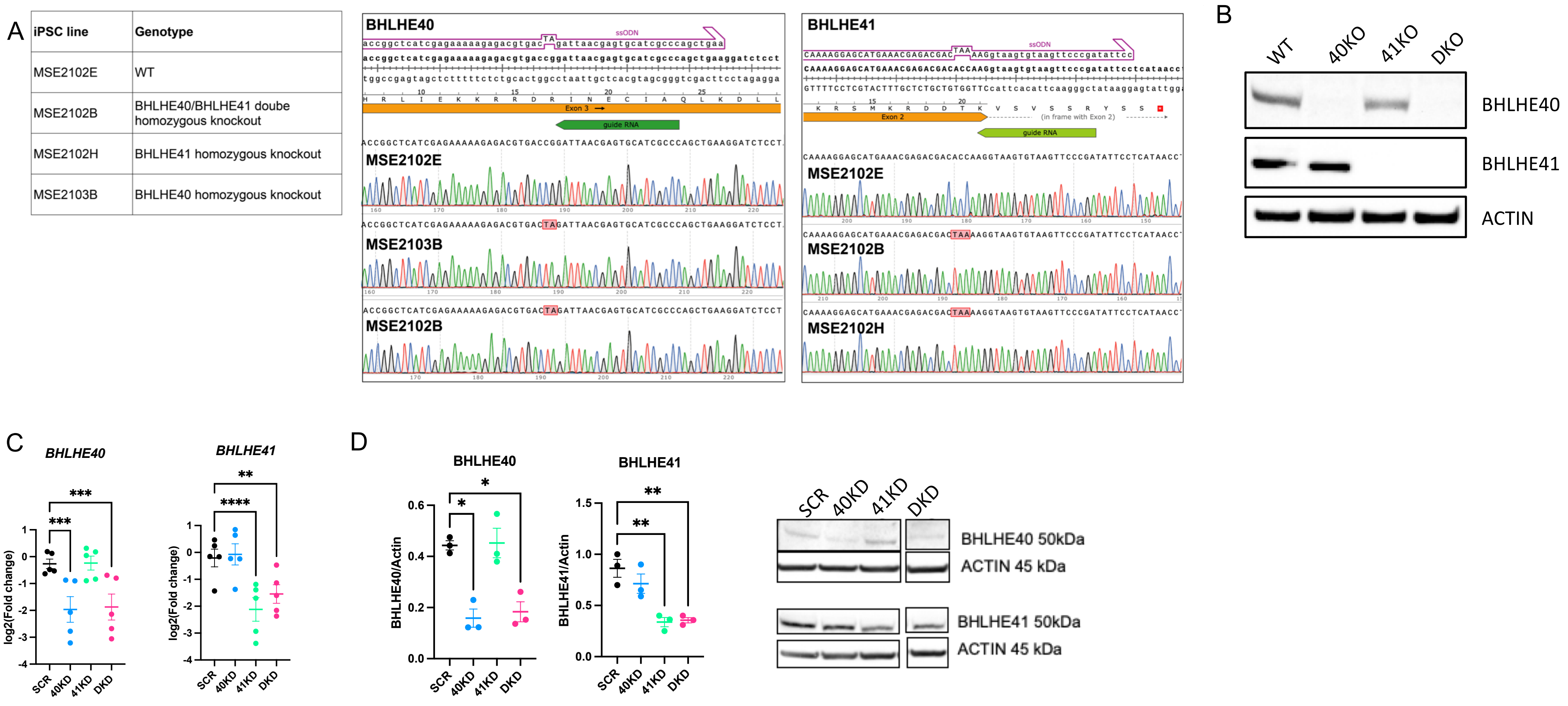

Supplementary Figure 3. Validation of knockout (KO) efficiency in human iPSC-derived microglia (iMGLs) with genetic inactivation of BHLHE40 and/or BHLHE41 and knockdown (KD) efficiency in human THP-1 macrophages (MACs) treated with BHLHE40 and/or BHLHE41 siRNAs. A) CRISPR/Cas9-mediated genome editing strategy to obtain homozygous BHLHE40 and/or BHLHE41 knockout human iPSC lines (Methods). **B)** Western blot confirming loss of BHLHE40 in 40KO and DKO iMGLs and loss of BHLHE41 in 41KO and DKO iMGLs. **C)** Expression of BHLHE40 and BHLHE41 measured by RT-qPCR, N=5/group. log2(fold change) (log2FC) is calculated with SCR MACs as reference. **D)** BHLHE40 and BHLHE41 normalized to Actin measured by western blot, N=3/group. Differences of means between groups were tested using one-way ANOVA with repeated measures followed by Dunnett's post-hoc test. * P-value < 0.05, ** P-value < 0.01, *** P-value < 0.001, **** P-value < 0.0001. Data plotted as mean ± SEM. Detailed statistics are shown in Supplementary File 1. 40KO = BHLHE40 KO iMGLs, 41KO = BHLHE41 KO iMGLs, DKO = BHLHE40 and BHLHE41 double KO iMGLs, WT = iMGLs derived from the parental iPSC line. 40KD = MACs treated with BHLHE40 siRNA, 41KD = MACs treated with BHLHE41 siRNA, DKD = MACs treated with BHLHE40 and BHLHE41 siRNA, SCR = MACs treated with scrambled siRNA.

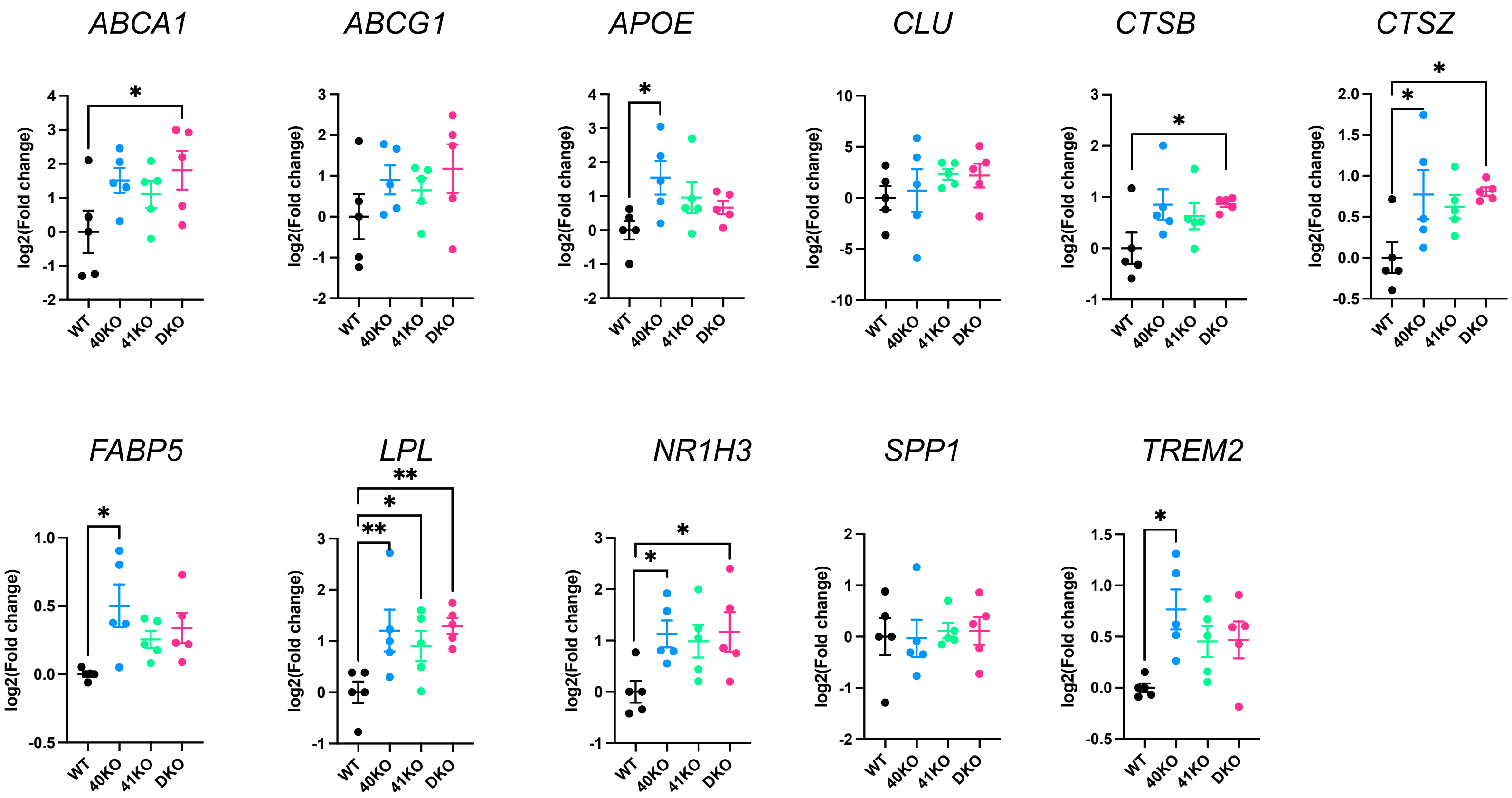

Supplementary Figure 4. Expression of lipid and lysosomal clearance genes in human iPSC-derived microglia (iMGLs) lacking *BHLHE40* and/or *BHLHE41*. Expression of lipid and lysosomal clearance genes measured by RT-qPCR, N=5/group. log2(fold change) (log2FC) is calculated with WT iMGLs as reference. Differences of means between groups were tested using one-way ANOVA with repeated measures followed by Dunnett's post-hoc test. * P-value < 0.05, ** P-value < 0.01. Data plotted as mean ± SEM. Detailed statistics are shown in Supplementary File 1. 40KO = BHLHE40 KO iMGLs, 41KO = BHLHE41 KO iMGLs, DKO = BHLHE40 and BHLHE41 double KO iMGLs, WT = iMGLs derived from the parental iPSC line.

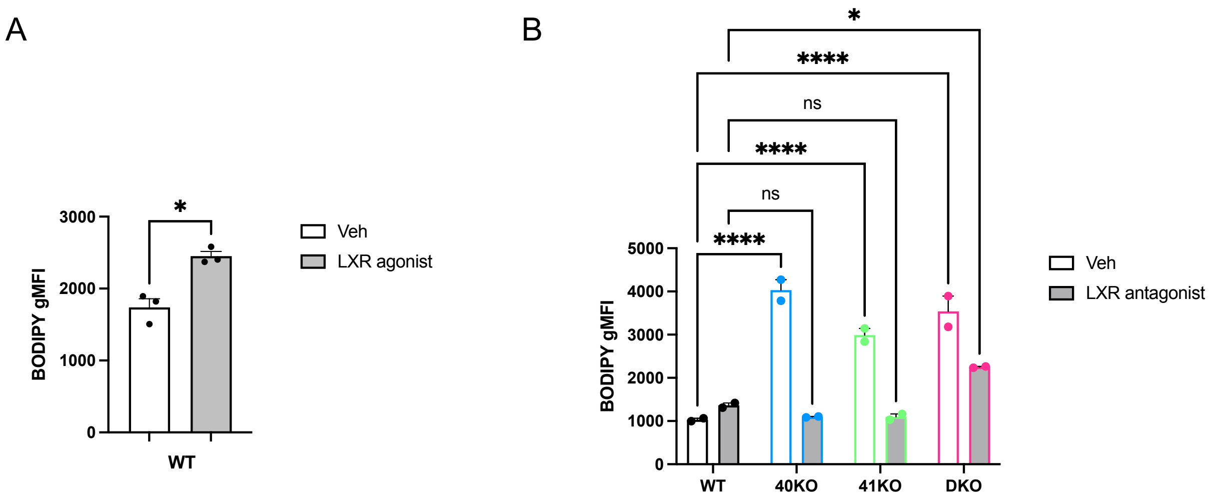

Supplementary Figure 5. LXR-dependent accumulation of lipid droplets (LDs) in human iPSC-derived microglia (iMGLs) lacking BHLHE40 and/or BHLHE41. A) LD content (BODIPY gMFI) measured by flow cytometry in BODIPY-positive WT iMGLs upon treatment with an LXR agonist (TO901317, 10uM, 48h) compared to vehicle control (Veh, DMSO), N=3/group. Differences of means between groups were tested using the paired t.test. * P-value < 0.05. **B)** LDs content (BODIPY gMFI) measured by flow cytometry in BODIPY-positive WT, 40KO, 41KO, and DKO iMGLs treated with an LXR antagonist (GSK2033, 2uM, 24h) compared to vehicle control (Veh, DMSO), N=2/group. Differences of means between groups were tested using two-way ANOVA followed by Dunnett's post-hoc tests. Data plotted as mean ± SEM. Detailed statistics are shown in Supplementary File 1. 40KO = BHLHE40 KO iMGLs, 41KO = BHLHE41 KO iMGLs, DKO = BHLHE40 and BHLHE41 double KO iMGLs, WT = iMGLs derived from the parental iPSC line.

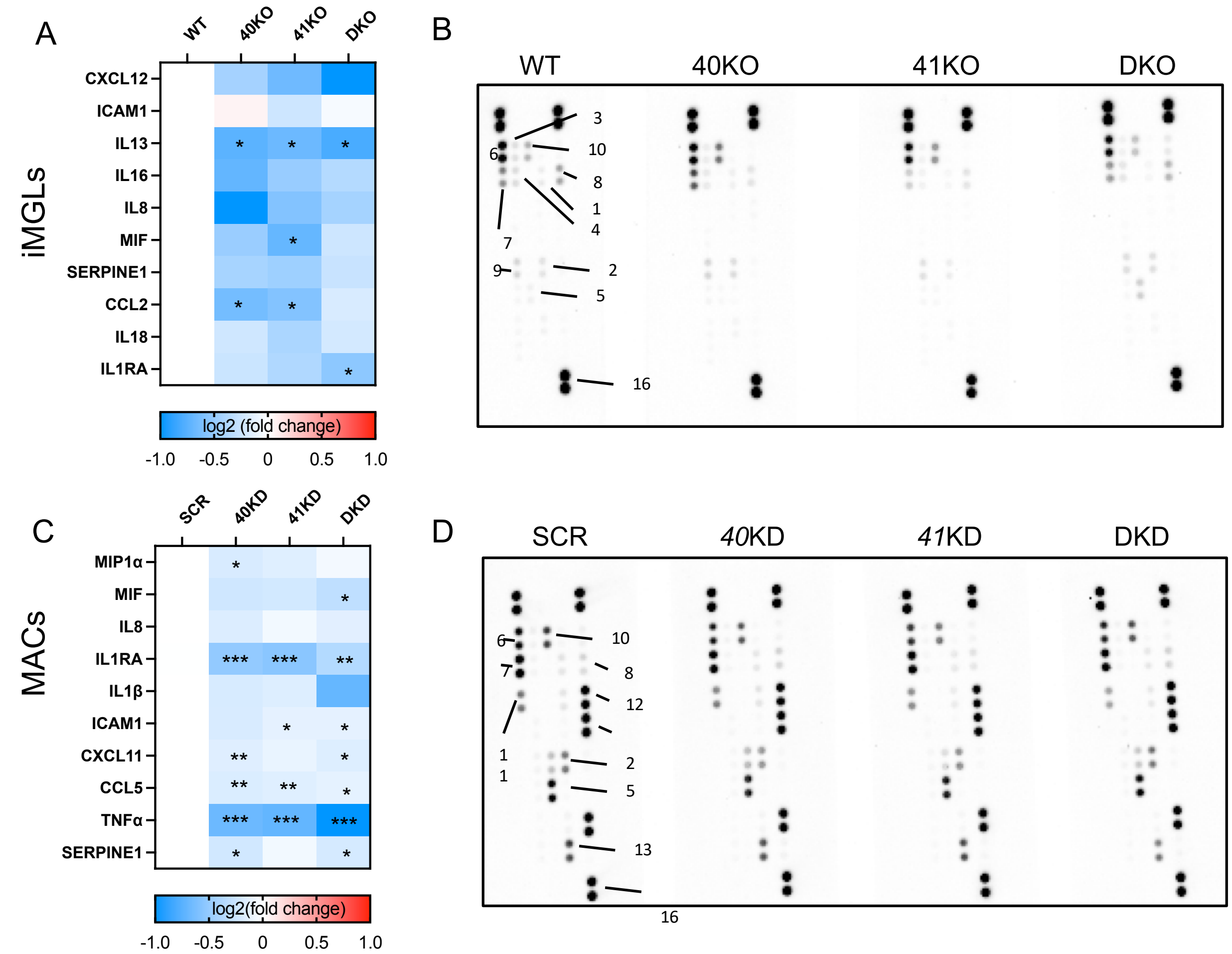

E

No.	Cytokine
1	CXCL12
2	ICAM1
3	IL13
4	IL16
5	IL8
6	MIF
7	SERPIN E1
8	CCL2
9	IL18
10	IL1RA
11	TNFα
12	MIP1α
13	IL1β
14	CCL5
15	CXCL11
16	Reference (ref)

Supplementary Figure 6. Reduce secretion of proinflammatory cytokines associated with complete loss (KO) or reduced levels (KD) of BHLHE40 and/or BHLHE41 in human iPSC-derived microglia (iMGLs) and THP-1 macrophages (MACs). Quantifications and dot blots of cytokines secreted in conditioned media from (A and B) human iPSC-derived microglia (iMGLs) with genetic inactivation of BHLHE40 and/or BHLHE41 or (C and D) human THP-1 macrophages (MACs) treated with BHLHE40 and/or BHLHE41 siRNAs measured using the Proteome Profiler Human Cytokine Array Kit (R&D Systems). **A and C)** Levels of cytokines secreted in conditioned media quantified as target density (i.e. mean target spot intensity multiplied by target spot area) normalized to reference density (i.e. mean reference spot intensity multiplied by reference spot area). log2(fold change) is calculated with (A) WT iMGLs or (C) SCR MACs as reference, N=3/group. **B and D)** Representative dot blots. **E)** Table of measured cytokines with corresponding dot identification number. Differences of means between groups were tested using one-way ANOVA with repeated measures followed by Dunnett's post-hoc test. *P-value<0.05, **P-value<0.01, ***P<0.001, ****P< 0.0001. Detailed statistics are shown in Supplementary File 1. 40KO = BHLHE40 KO iMGLs, 41KO = BHLHE41 KO iMGLs, DKO = BHLHE40 and BHLHE41 double KO iMGLs, WT = iMGLs derived from the parental iPSC line. 40KD = MACs treated with BHLHE40 siRNA, 41KD = MACs treated with BHLHE41 siRNA, DKD = MACs treated with BHLHE40 and BHLHE41 siRNA, SCR = MACs treated with scrambled siRNA.

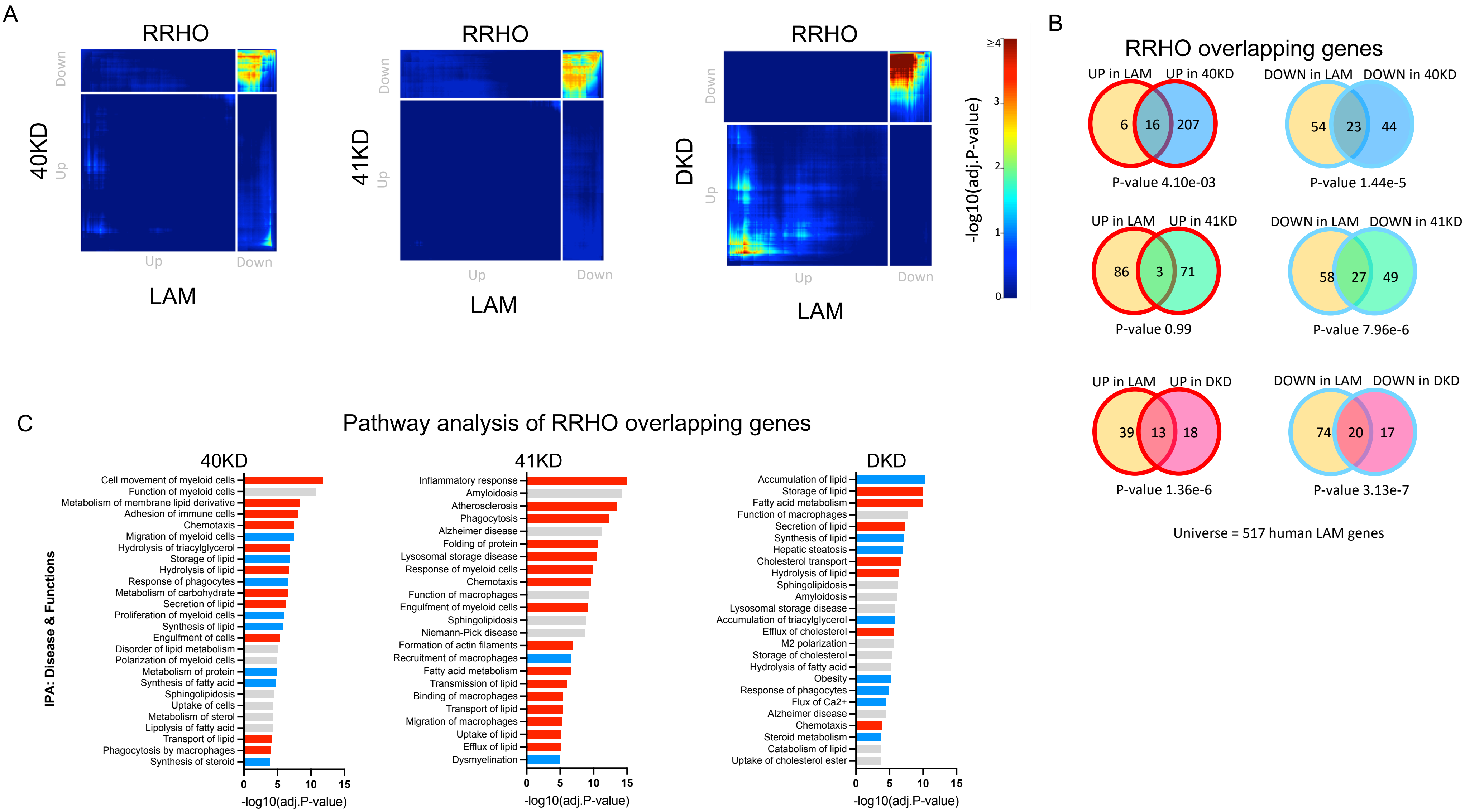
A
RRHO
40KD
LAM
41KD
DKD
Up
Down
-log10(adj.P-value)
≥4
3
2
1
0
B
RRHO overlapping genes
UP in LAM
UP in 40KD
6
16
207
P-value 4.10e-03
DOWN in LAM
DOWN in 40KD
54
23
44
P-value 1.44e-5
UP in 41KD
86
3
71
P-value 0.99
DOWN in 41KD
58
27
49
P-value 7.96e-6
UP in DKD
39
13
18
P-value 1.36e-6
DOWN in DKD
74
20
17
P-value 3.13e-7
Universe = 517 human LAM genes
C
Pathway analysis of RRHO overlapping genes
IPA: Disease & Functions
40KD
Cell movement of myeloid cells
Function of myeloid cells
Metabolism of membrane lipid derivative
Adhesion of immune cells
Chemotaxis
Migration of myeloid cells
Hydrolysis of triacylglycerol
Storage of lipid
Hydrolysis of lipid
Response of phagocytes
Metabolism of carbohydrate
Secretion of lipid
Proliferation of myeloid cells
Synthesis of lipid
Engulfment of cells
Disorder of lipid metabolism
Polarization of myeloid cells
Metabolism of protein
Synthesis of fatty acid
Sphingolipidosis
Uptake of cells
Metabolism of sterol
Lipolysis of fatty acid
Transport of lipid
Phagocytosis by macrophages
Synthesis of steroid
41KD
Inflammatory response
Amyloidosis
Atherosclerosis
Phagocytosis
Alzheimer disease
Folding of protein
Lysosomal storage disease
Response of myeloid cells
Chemotaxis
Function of macrophages
Engulfment of myeloid cells
Sphingolipidosis
Niemann-Pick disease
Formation of actin filaments
Recruitment of macrophages
Fatty acid metabolism
Transmission of lipid
Binding of macrophages
Transport of lipid
Migration of macrophages
Uptake of lipid
Efflux of lipid
Dysmyelination
DKD
Accumulation of lipid
Storage of lipid
Fatty acid metabolism
Function of macrophages
Secretion of lipid
Synthesis of lipid
Hepatic steatosis
Cholesterol transport
Hydrolysis of lipid
Sphingolipidosis
Amyloidosis
Lysosomal storage disease
Accumulation of triacylglycerol
Efflux of cholesterol
M2 polarization
Storage of cholesterol
Hydrolysis of fatty acid
Obesity
Response of phagocytes
Flux of Ca2+
Alzheimer disease
Chemotaxis
Steroid metabolism
Catabolism of lipid
Uptake of cholesterol ester
0
5
10
15
-log10(adj.P-value)

Supplementary Figure 7. Knockdown of BHLHE40/41 partially recapitulates the LAM transcriptional response in human THP-1 macrophages (MACs). A) Rank-rank hypergeometric overlap (RRHO) heatmaps visualizing significant overlaps in gene expression changes between each pair of BHLHE40/41 knockdown (KD) MAC and human LAM (Jaitin et al. [3], Dataset S6, FDR Adj.P-value < 0.05) transcriptional signatures. Adj.P-value in color temperature scale represents Benjamini-Hochberg corrected P-value of hypergeometric overlap test. **B)** Venn diagrams of most significant overlaps between genes up-regulated in both *BHLHE40/41* KD MAC and human LAM transcriptional signatures, corresponding to the warmest pixel in the bottom-left quadrant of the respective heatmap (left) and Venn diagrams of most significant overlaps between genes down-regulated in both BHLHE40/41 KD MAC and human LAM transcriptional signatures, corresponding to the warmest pixel in the upper-right quadrant of the respective heatmap (right). P-values are calculated using the hypergeometric overlap test restricted to the universe of 517 human LAM genes (Jaitin *et al*. [3], Dataset S6, FDR Adj.P-value < 0.05). **C)** Pathways ("diseases and biological functions" category) found by IPA to be significantly enriched for RRHO overlapping genes. Red bars represent pathways predicted by IPA to be more active (i.e., with positive Z-scores), blue bars represent pathways predicted by IPA to be less active (i.e., with negative Z-scores), grey bars represent pathways with non-attributed Z-scores. Adj.P-value on the x-axis represents Bonferroni-Holm corrected P-value of pathway enrichment test. 40KD = MACs treated with BHLHE40 siRNA, 41KD = MACs treated with BHLHE41 siRNA, DKD = MACs treated with BHLHE40 and BHLHE41 siRNA, SCR = MACs treated with scrambled siRNA.

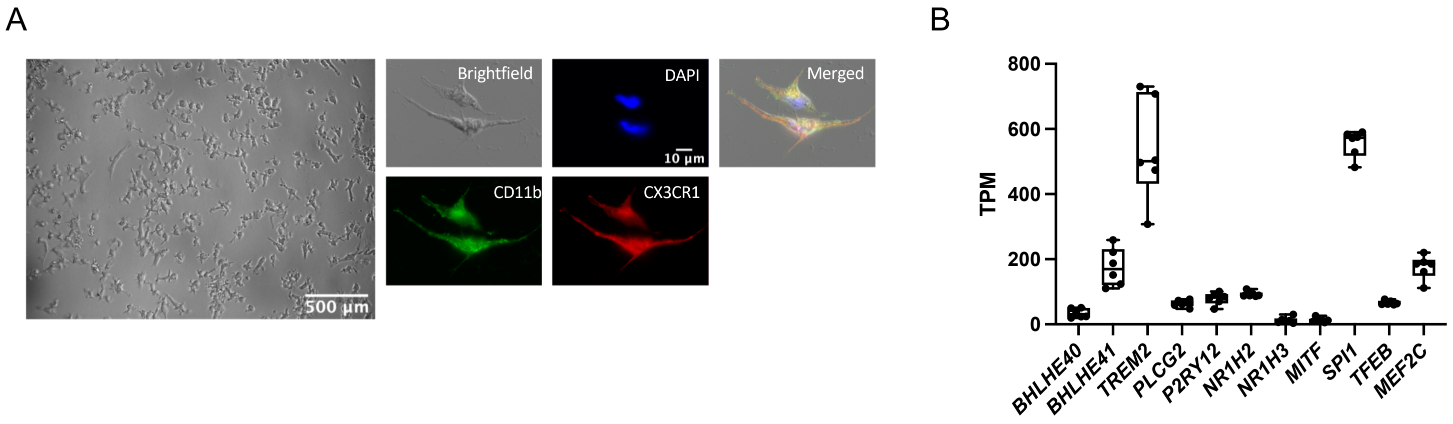

Supplementary Figure 8. **Differentiation of human iPSC lines into microglia-like cells (iMGLs). A)** Representative bright-field microscopic images and immunofluorescence staining for nuclei (DAPI) and differentiation markers (CD11b and CX3CR1) of a typical culture of mature iMGLs after 25d *in vitro*. **B)** Expression of *BHLHE40* and *BHLHE41* and other microglial genes (*P2RY12*, *PLCG2*, and *TREM2*) and transcription factors (*SPI1*, *MITF*, *MEF2C*, *NR1H2*, *NR1H3*, and *TFEB*) of interest measured by RNA-seq in iMGLs derived from the parental iPSC line, N=5/group. TPM = transcripts per million.

bioRxiv preprint doi: https://doi.org/10.1101/2023.02.13.528372; this version posted February 13, 2023. The copyright holder for this preprint (which was not certified by peer review) is the author/funder, who has granted bioRxiv a license to display the preprint in perpetuity. It is made available under aCC-BY 4.0 International license.

Figure legends with detailed statistical tests used in Figure 5, Figure 6, Supplementary Figure 3, Supplementary Figure 4, Supplementary Figure 5, Supplementary Figure 6

Figure 5. Knockout of BHLHE40/41 increases expression of lipid and lysosomal clearance genes, cholesterol efflux and lipid droplet content, lysosomal mass and degradative capacity in human iPSC-derived microglia (iMGLs).

A. **Expression of lipid and lysosomal clearance genes measured by RT-qPCR.** from N=5 independent iMGL differentiations/group (separate plots and statistical details for each gene in Supplementary Figure 5 and in the legend for Supplementary Figure 5 below). Post-hoc tests * P-value < 0.05, ** P-value < 0.01
B. **Intracellular APOE normalized to Actin measured by western blot.** Differences of means between groups were tested with one-way repeated measures ANOVA F(3,6)=10.66, P-value=0.0081, N=3 independent iMGL differentiations/group, followed by Dunnett's post-hoc tests: $ES_{40KO-WT}$=1.04 [0.43, 1.68], $\text{P-value}_{40KO-WT}$=0.0050; $ES_{41KO-WT}$=0.46 [-0.48, 0.77], $\text{P-value}_{41KO-WT}$=0.8204; ES_{DKO-WT}=0.98 [-0.28, 0.97], P-value_{DKO-WT}=0.2906.
C. **ABCA1 normalized to Actin measured by western blot.** Differences of means between groups were tested with one-way repeated measures ANOVA F(3,6)=13.11, P-value=0.0048, N=3 independent iMGL differentiations/group, followed by Dunnett's post-hoc tests: $ES_{40KO-WT}$=0.17 [-0.04, 0.38], $\text{P-value}_{40KO-WT}$=0.1068; $ES_{41KO-WT}$=0.42 [0.21, 0.63], $\text{P-value}_{41KO-WT}$=0.0021; ES_{DKO-WT}=0.14 [-0.06, 0.35], P-value_{DKO-WT}=0.1701.
D. **Secreted APOE normalized to total protein measured by ELISA.** Differences of means between groups were tested with one-way repeated measures ANOVA F(3,9)=6.41, P-value=0.0130, N=5 independent iMGL differentiations/group, followed by Dunnett's post-hoc tests: $ES_{40KO-WT}$=5.76 [2.03, 9.49], $\text{P-value}_{40KO-WT}$=0.0048; $ES_{41KO-WT}$=3.53 [-0.19, 7.23], $\text{P-value}_{41KO-WT}$=0.0630; ES_{DKO-WT}=2.93 [-0.79, 6.66], P-value_{DKO-WT}=0.1271.

bioRxiv preprint doi: https://doi.org/10.1101/2023.02.13.528372; this version posted February 13, 2023. The copyright holder for this preprint (which was not certified by peer review) is the author/funder, who has granted bioRxiv a license to display the preprint in perpetuity. It is made available under aCC-BY 4.0 International license.

E. **Percentage of cholesterol efflux.** Differences of means between groups were tested with one-way repeated measures ANOVA F(3,12)=24.68, P-value<0.0001, N=6 independent iMGL differentiations/group, followed by Dunnett's post-hoc tests: $ES_{40KO-WT}$=6.80 [3.76, 9.84], P-$value_{40KO-WT}$=0.002; $ES_{41KO-WT}$=7.80 [4.76, 10.84], P-$value_{41KO-WT}$<0.0001; ES_{DKO-WT}=8.80 [5.76, 11.84], P-$value_{DKO-WT}$<0.0001.

F. **LD content (BODIPY gMFI) measured by flow cytometry in BODIPY-positive cells** (top). Differences of means between groups were tested with one-way repeated measures ANOVA F(3,15)=7.23, P-value=0.0032, N=6 independent iMGL differentiations/group, followed by Dunnett's post-hoc tests: $ES_{40KO-WT}$=1928 [783.7, 3072], P-$value_{40KO-WT}$=0.0014; $ES_{41KO-WT}$=1151 [7.441, 2295], P-$value_{41KO-WT}$=0.0484; ES_{DKO-WT}=1546 [401.8, 2690], P-$value_{DKO-WT}$=0.0082. Representative flow-cytometry histograms (bottom)

G. **Lysosomal mass (LysoTracker gMFI) measured by flow cytometry in LysoTracker-positive cells** (top). Differences of means between groups were tested with one-way repeated measures ANOVA F(3,12)=2.81, P-value=0.0845, N=5 independent iMGL differentiations/group, followed by Dunnett's post-hoc tests: $ES_{40KO-WT}$=1987 [-1982, 5957], P-$value_{40KO-WT}$=0.4270; $ES_{41KO-WT}$=3125 [-844.9, 7094], P-$value_{41KO-WT}$=0.1341; ES_{DKO-WT}=4080 [109.9, 8049], P-$value_{DKO-WT}$=0.0438. Representative flow-cytometry histograms (bottom)

H. **Lysosomal acidification (LysoSensor gMFI) measured by flow cytometry in LysoSensor-positive cells** (top). Differences of means between groups were tested with one-way repeated measures ANOVA F(3,12)=4.41, P-value=0.0261, N=5 independent iMGL differentiations/group, followed by Dunnett's post-hoc tests: $ES_{40KO-WT}$=1327 [-19426, 22080], P-$value_{40KO-WT}$=0.9963; $ES_{41KO-WT}$=2858 [-17895, 23611], P-$value_{41KO-WT}$=0.9661; ES_{DKO-WT}=24256 [3502, 45009], P-$value_{DKO-WT}$=0.0222. Representative flow-cytometry histograms (bottom)

I. **Lysosomal proteolysis (DQ-BSA gMFI) measured by flow cytometry in DQ-BSA-positive cells** (top). Differences of means between groups were tested with one-way repeated measures ANOVA F(3,12)=9.48, P-value=0.0017, N=5 independent iMGL differentiations/group, followed by Dunnett's post-hoc tests:

bioRxiv preprint doi: https://doi.org/10.1101/2023.02.13.528372; this version posted February 13, 2023. The copyright holder for this preprint (which was not certified by peer review) is the author/funder, who has granted bioRxiv a license to display the preprint in perpetuity. It is made available under aCC-BY 4.0 International license.

$ES_{40KO-WT}$=3846 [-961.2, 8653], P-$value_{40KO-WT}$=0.1265; $ES_{41KO-WT}$=3125 [5.313, 9620], P-$value_{41KO-WT}$=0.0497; ES_{DKO-WT}=9485 [4678, 14293], P-$value_{DKO-WT}$=0.0005. Representative flow-cytometry histograms (bottom).

J. **DQ-BSA red fluorescent signal (total integrated density) measured over time using the Incucyte S3 live imaging system.** Differences of means between groups were tested with two-way repeated measures ANOVA F(3.8)=13.93, P-value=0.0015, N=3 independent iMGL differentiation/group, followed by Dunnett's post-hoc tests: $ES_{40KO-WT}$=37.34 [-106.7, 181.4], P-$value_{40KO-WT}$=0.7997; $ES_{41KO-WT}$=192 [47.91, 336.0], P-$value_{41KO-WT}$=0.0126; ES_{DKO-WT}=281.6 [137.5, 425.6], P-$value_{DKO-WT}$=0.0013. Total integrated density was calculated as mean red fluorescent intensity multiplied by surface area of masked object (i.e. cell), [RCU x μm^2].

Effect sizes (ES) are reported as unstandardized point estimates with 95% confidence intervals in the same unit as depicted on each graph.

Figure 6. Knockdown of BHLHE40/41 increases expression of lipid and lysosomal clearance genes, cholesterol efflux and lipid droplet content in human THP1 macrophages (MACs).

A. **Expression of lipid and lysosomal clearance genes measured by RT-qPCR.** from N=7 independent MAC differentiation (transient transfection with siRNA)/group. Post-hoc tests * P-value < 0.05, ** P-value < 0.01
B. **Secreted APOE normalized to total protein measured by ELISA.** Differences of means between groups were tested with one-way repeated measures ANOVA F(3,15)=2.52, P-value=0.0976, N=6 independent MAC differentiations/transient transfection with siRNA per group, followed by Dunnett's post-hoc tests: $ES_{40KD-SCR}$=2.33 [0.04, 4.62], P-$value_{40KO-WT}$=0.0457; $ES_{41KD-SCR}$=0.66 [-1.62, 2.94], P-$value_{41KD-SCR}$=0.7914; $ES_{DKD-SCR}$=0.8324 [1.45, 3.12], P-$value_{DKD-SCR}$=0.6689.
C. **Percentage of cholesterol efflux.** Differences of means between groups were tested with one-way repeated measures ANOVA F(3,15)=22.82, P-value<0.0001,

bioRxiv preprint doi: https://doi.org/10.1101/2023.02.13.528372; this version posted February 13, 2023. The copyright holder for this preprint (which was not certified by peer review) is the author/funder, who has granted bioRxiv a license to display the preprint in perpetuity. It is made available under aCC-BY 4.0 International license.

N=6 independent MAC differentiations/transient transfection with siRNA per group, followed by Dunnett's post-hoc tests: $ES_{40KD-SCR}$=12.45 [8.47, 16.44], P-value$_{40KO-WT}$<0.0001; $ES_{41KD-SCR}$=4.83 [0.84, 8.81], P-value$_{41KD-SCR}$=0.0170; $ES_{DKD-SCR}$=6.906 [2.92, 10.89], P-value$_{DKD-SCR}$=0.0011.

D. **LD content (BODIPY gMFI) measured by flow cytometry in BODIPY-positive cells.** Differences of means between groups were tested with one-way repeated measures ANOVA F(3,15)=10.31, P-value=0.0006, N=6 independent MAC differentiations/transient transfection with siRNA per group, followed by Dunnett's post-hoc tests: $ES_{40KD-SCR}$=2348 [567.0, 4128], P-value$_{40KO-WT}$=0.0097; $ES_{41KD-SCR}$=2890 [1110, 4671], P-value$_{41KD-SCR}$=0.0020; $ES_{DKD-SCR}$=3563 [1782, 5343], P-value$_{DKD-SCR}$=0.0003. Gates were drown based on fluorescence minus one (FMO) controls.

Effect sizes (ES) are reported as unstandardized point estimates with 95% confidence intervals in the same unit as depicted on each graph.

Supplementary Figure 3. Validation of knockout (KO) efficiency in human iPSC-derived microglia (iMGLs) with genetic inactivation of BHLHE40 and/or BHLHE41 and knockdown (KD) efficiency in human THP-1 macrophages (MACs) treated with BHLHE40 and/or BHLHE41 siRNAs.

A. CRISPR-Cas9 genome editing strategy to obtain homozygous BHLHE40 and/or BHLHE41 knockout human iPSC lines.
B. Western blot confirming loss of BHLHE40 in 40KO and DKO iMGLs and loss of BHLHE41 in 41KO and DKO iMGLs.
C. **Expression of BHLHE40 and BHLHE41 measured by RT-qPCR, N=5/group. log2(fold change) (log2FC) is calculated with SCR MACs as reference.** Differences of means between groups were tested with one-way repeated measures followed by Dunnett's post-hoc tests

   BHLHE40: ANOVA F(3,12)=20.02, P-value<0.0001, N=5 independent MAC differentiations/transient transfection with siRNA per group, followed by Dunnett's post-hoc tests: $ES_{40KD-SCR}$=-1.699 [-2.498, -0.8999], P-value$_{40KO-WT}$=0.0003;

bioRxiv preprint doi: https://doi.org/10.1101/2023.02.13.528372; this version posted February 13, 2023. The copyright holder for this preprint (which was not certified by peer review) is the author/funder, who has granted bioRxiv a license to display the preprint in perpetuity. It is made available under aCC-BY 4.0 International license.

$ES_{41KD-SCR}$=0.0283 [-0.7707, 0.8274], P-$value_{41KD-SCR}$=0.9993; $ES_{DKD-SCR}$=-1.606 [-2.405, -0.8073], P-$value_{DKD-SCR}$=0.0005.

BHLHE41: ANOVA F(3,12)=25.21, P-value<0.0001, N=5 independent MAC differentiations/transient transfection with siRNA per group, followed by Dunnett's post-hoc tests: $ES_{40KD-SCR}$=0.1356 [-0.6250, 0.8963], P-$value_{40KO-WT}$=0.9318; $ES_{41KD-SCR}$=1.912 [-2.673, 1.151], P-$value_{41KD-SCR}$<0.0001; $ES_{DKD-SCR}$=-1.338 [-2.098, -0.5770,], P-$value_{DKD-SCR}$=0.0014.

D. **BHLHE40 and BHLHE41 normalized to Actin measured by western blot**

Differences of means between groups were tested with one-way repeated measures followed by Dunnett's post-hoc tests

BHLHE40*:* ANOVA F(3,6)=12.51, P-value<0.0054, N=3 independent MAC differentiations/transient transfection with siRNA per group, followed by Dunnett's post-hoc tests: $ES_{40KD-SCR}$=-0.2847 [-0.4832, 0.0862], P-$value_{40KO-WT}$=0.0107; $ES_{41KD-SCR}$=-0.0091 [-0.1894, 0.2076], P-$value_{41KD-SCR}$=0.9977; $ES_{DKD-SCR}$=-0.2599 [-0.4584, -0.0613], P-$value_{DKD-SCR}$=0.0162.

BHLHE41*:* ANOVA F(3,12)=14.62, P-value=0.0036, N=3 independent MAC differentiations/transient transfection with siRNA per group, followed by Dunnett's post-hoc tests: $ES_{40KD-SCR}$=-0.1502 [-0.4509, 0.1504], P-$value_{40KO-WT}$=0.3576; $ES_{41KD-SCR}$=-0.5257 [-0.8264, -0.2250], P-$value_{41KD-SCR}$=0.0041; $ES_{DKD-SCR}$=-0.5075 [-0.8082, -0.2069], P-$value_{DKD-SCR}$=0.0048.

Effect sizes (ES) are reported as unstandardized point estimates with 95% confidence intervals in the same unit as depicted on each graph.

Supplementary Figure 4. Expression of lipid and lysosomal clearance genes in human iPSC-derived microglia (iMGLs) lacking *BHLHE40* and/or *BHLHE41*. Expression of lipid and lysosomal clearance genes measured by RT-qPCR, N=5/group. log2(fold change) (log2FC) is calculated with WT iMGLs as reference. Differences of means between groups were tested using one-way ANOVA with repeated measures followed by Dunnett's post-hoc test.

bioRxiv preprint doi: https://doi.org/10.1101/2023.02.13.528372; this version posted February 13, 2023. The copyright holder for this preprint (which was not certified by peer review) is the author/funder, who has granted bioRxiv a license to display the preprint in perpetuity. It is made available under aCC-BY 4.0 International license.

ABCA1: ANOVA F(3,12)=3.17, P-value=0.0640 followed by Dunnett's post-hoc tests: $ES_{40KO\text{-}WT}$=1.51 [-0.1786, 3.207], $P\text{-value}_{40KO\text{-}WT}$=0.0822; $ES_{41KO\text{-}WT}$=1.102 [-0.5912, 2.794], $P\text{-value}_{41KO\text{-}WT}$=0.2408; $ES_{DKO\text{-}WT}$=1.813 [0.1202, 3.506], $P\text{-value}_{DKO\text{-}WT}$=0.0356.

ABCG1: ANOVA F(3,12)=1.45, P-value=0.2783 followed by Dunnett's post-hoc tests: $ES_{40KO\text{-}WT}$=0.8994 [-0.6879, 2.487], $P\text{-value}_{40KO\text{-}WT}$=0.3357; $ES_{41KO\text{-}WT}$=0.6468 [-0.9405, 2.234], $P\text{-value}_{41KO\text{-}WT}$=0.5775; $ES_{DKO\text{-}WT}$=1.178 [-0.4097, 2.765], $P\text{-value}_{DKO\text{-}WT}$=0.1637.

APOE: ANOVA F(3,12)=3.89, P-value=0.0372 followed by Dunnett's post-hoc tests: $ES_{40KO\text{-}WT}$=1.546 [0.3100, 2.782], $P\text{-value}_{40KO\text{-}WT}$=0.0149; $ES_{41KO\text{-}WT}$=0.9622 [-0.2740, 2.198], $P\text{-value}_{41KO\text{-}WT}$=0.1395; $ES_{DKO\text{-}WT}$=0.6654 [-0.5708, 1.902], $P\text{-value}_{DKO\text{-}WT}$=0.3732.

CLU: ANOVA F(3,12)=0.7083, P-value=0.5654 followed by Dunnett's post-hoc tests: $ES_{40KO\text{-}WT}$=0.7216 [-4.371, 5.814], $P\text{-value}_{40KO\text{-}WT}$=0.9633; $ES_{41KO\text{-}WT}$=2.306 [-2.786, 7.399], $P\text{-value}_{41KO\text{-}WT}$=0.5016; $ES_{DKO\text{-}WT}$=2.189 [-2.903, 7.282], $P\text{-value}_{DKO\text{-}WT}$=0.5395.

CTSB: ANOVA F(3,12)=2.620, P-value=0.0988 followed by Dunnett's post-hoc tests: $ES_{40KO\text{-}WT}$=0.8508 [-0.099, 1.801], $P\text{-value}_{40KO\text{-}WT}$=0.0818; $ES_{41KO\text{-}WT}$=0.6270 [-0.3231, 1.577], $P\text{-value}_{41KO\text{-}WT}$=0.2319; $ES_{DKO\text{-}WT}$=0.8646 [-0.0548, 1.815], $P\text{-value}_{DKO\text{-}WT}$=0.0764.

CTSZ: ANOVA F(3,12)=4.201, P=0.0301 followed by Dunnett's post-hoc tests: $ES_{40KO\text{-}WT}$=0.7712 [0.0751, 1.467], $P\text{-value}_{40KO\text{-}WT}$=0.0298; $ES_{41KO\text{-}WT}$=0.6248 [-0.0713, 1.321], $P\text{-value}_{41KO\text{-}WT}$=0.0810; $ES_{DKO\text{-}WT}$=0.8092 [0.1131, 1.505], $P\text{-value}_{DKO\text{-}WT}$=0.0229.

FABP5: ANOVA F(3,12)=3.720, P-value=0.0422 followed by Dunnett's post-hoc tests: $ES_{40KO\text{-}WT}$=0.5012 [0.0895, 0.9129], $P\text{-value}_{40KO\text{-}WT}$=0.0175; $ES_{41KO\text{-}WT}$=0.2558 [-0.1559, 0.6675], $P\text{-value}_{41KO\text{-}WT}$=0.2712; $ES_{DKO\text{-}WT}$=0.3398 [-0.0719, 0.7515], $P\text{-value}_{DKO\text{-}WT}$=0.1129.

LPL: ANOVA F(3,12)=7.025, P-value=0.0056 followed by Dunnett's post-hoc tests: $ES_{40KO\text{-}WT}$=1.207 [0.3591, 2.054], $P\text{-value}_{40KO\text{-}WT}$=0.0065; $ES_{41KO\text{-}WT}$=0.9020 [0.0544, 1.750], $P\text{-value}_{41KO\text{-}WT}$=0.0368; $ES_{DKO\text{-}WT}$=1.296 [0.4487, 2.144], $P\text{-value}_{DKO\text{-}WT}$=0.0039.

NR1H3: ANOVA F(3,12)=3.438, P-value=0.0520 followed by Dunnett's post-hoc tests: $ES_{40KO\text{-}WT}$=1.129 [0.001, 2.258], $P\text{-value}_{40KO\text{-}WT}$=0.0500; $ES_{41KO\text{-}WT}$=0.9856 [-0.1442, 2.115], $P\text{-value}_{41KO\text{-}WT}$=0.0910; $ES_{DKO\text{-}WT}$=1.166 [0.0364, 2.296], $P\text{-value}_{DKO\text{-}WT}$=0.0429.

bioRxiv preprint doi: https://doi.org/10.1101/2023.02.13.528372; this version posted February 13, 2023. The copyright holder for this preprint (which was not certified by peer review) is the author/funder, who has granted bioRxiv a license to display the preprint in perpetuity. It is made available under aCC-BY 4.0 International license.

SPP1: ANOVA $F(3,12)=0.0833$, P-value=0.9678 followed by Dunnett's post-hoc tests: $ES_{40KO\text{-}WT}=-0.0308$ [-1.027, 0.9655], $P_{40KO\text{-}WT}=0.9996$; $ES_{41KO\text{-}WT}=0.1156$ [-0.8807, 1.112], $P\text{-value}_{41KO\text{-}WT}=0.9790$; $ES_{DKO\text{-}WT}=0.1126$ [-0.8837, 1.109], $P\text{-value}_{DKO\text{-}WT}=0.9805$.

TREM2: ANOVA $F(3,12)=4.113$, P-value=0.0320 followed by Dunnett's post-hoc tests: $ES_{40KO\text{-}WT}=0.7654$ [0.1746, 1.356], $P\text{-value}_{40KO\text{-}WT}=0.0120$; $ES_{41KO\text{-}WT}=0.4536$ [-0.1372, 1.044], $P\text{-value}_{41KO\text{-}WT}=0.1462$; $ES_{DKO\text{-}WT}=0.4688$ [-0.1220, 1.060], $P\text{-value}_{DKO\text{-}WT}=0.1303$.

Effect sizes (ES) are reported as unstandardized point estimates with 95% confidence intervals in the same unit as depicted on each graph.

Supplementary Figure 5. LXR-dependent accumulation of lipid droplets (LDs) in human iPSC-derived microglia (iMGLs) lacking BHLHE40 and/or BHLHE41.

A. LD content (BODIPY gMFI) measured by flow cytometry in BODIPY-positive WT iMGLs upon treatment with an LXR agonist (TO901317, 10uM, 48h) compared to vehicle control (Veh, DMSO). Differences of means between groups were tested using the paired t.test. t=7.049, df=2, P-value=0.0195, N=3 independent iMGLs differentiations per group $ES_{LXR\_agonist\text{-}Veh}=711.3$ [277.2, 1145]
B. LD content (BODIPY gMFI) measured by flow cytometry in BODIPY-positive WT, 40KO, 41KO, and DKO iMGLs treated with an LXR antagonist (GSK2033, 2uM, 24h) compared to vehicle control (Veh, DMSO). Differences of means between groups were tested using two-way ANOVA Genotype: $F(3,8)=34.17$, $P<0.0001$, Treatment: $F(1,8)=152.9$, P-value<0.0001, Genotype x Treatment $F(3,8) = 40.04$, P-value<0.0001, N=2 independent iMGLs differentiation followed by Dunnett's post-hoc tests Veh: $ES_{40KO\text{-}WT}=2999$ [2325, 3672], $P\text{-value}_{40KO\text{-}WT}<0.0001$; $ES_{41KO\text{-}WT}=1959$ [1286, 2632], $P\text{-value}_{41KO\text{-}WT}<0.0001$; $ES_{DKO\text{-}WT}=2508$ [1835, 3181], $P\text{-value}_{DKO\text{-}WT}<0.0001$; LXR antagonist: $ES_{40KO\text{-}WT}=-268.2$ [-941.1, 405.1], $P\text{-value}_{40KO\text{-}WT}=0.5531$; $ES_{41KO\text{-}WT}=-265.4$ [-938.6, 407.8], $P\text{-value}_{41KO\text{-}WT}=0.5602$; $ES_{DKO\text{-}WT}=887.1$ [213.8, 1560], $P\text{-value}_{DKO\text{-}WT}=0.0133$

bioRxiv preprint doi: https://doi.org/10.1101/2023.02.13.528372; this version posted February 13, 2023. The copyright holder for this preprint (which was not certified by peer review) is the author/funder, who has granted bioRxiv a license to display the preprint in perpetuity. It is made available under aCC-BY 4.0 International license.

Data plotted as mean ± SEM. 40KO = BHLHE40 KO iMGLs, 41KO = BHLHE41 KO iMGLs, DKO = BHLHE40 and BHLHE41 double KO iMGLs, WT = iMGLs derived from the parental iPSC line. Effect sizes (ES) are reported as unstandardized point estimates with 95% confidence intervals in the same unit as depicted on each graph

Supplementary Figure 6. Reduced secretion of proinflammatory cytokines associated with complete loss (KO) or reduced levels (KD) of BHLHE40 and/or BHLHE41 in human iPSC-derived microglia (iMGLs) and THP-1 macrophages (MACs). Quantifications and dot blots of cytokines secreted in conditioned media from (A and B) human iPSC-derived microglia (iMGLs) with genetic inactivation of BHLHE40 and/or BHLHE41 or (C and D) human THP-1 macrophages (MACs) treated with BHLHE40 and/or BHLHE41 siRNAs measured using the Proteome Profiler Human Cytokine Array Kit (R&D Systems).

A. Levels of cytokines secreted in conditioned media quantified as target density (i.e. mean target spot intensity multiplied by target spot area) normalized to reference density (i.e. mean reference spot intensity multiplied by reference spot area). Data are plotted as log2(fold change) which is calculated with WT iMGLs. Differences of means between groups were tested using one-way ANOVA with repeated measures followed by Dunnett's post-hoc test. N=3/group

CXCL12: ANOVA F(3,6)=2.42, P-value=0.1645, followed by Dunnett's post-hoc tests: $ES_{40KO\text{-}WT}$=-0.0181 [-0.2717, 0.2353], P-$value_{40KO\text{-}WT}$=0.9916; $ES_{41KO\text{-}WT}$=-0.0817 [-0.3352, 0.1718], P-$value_{41KO\text{-}WT}$=0.6498; $ES_{DKO\text{-}WT}$=-0.1990 [-0.4525, 0.0545], P-$value_{DKO\text{-}WT}$=0.1163.

ICAM1: ANOVA F(3,6)=0.745, P-value=0.5634, followed by Dunnett's post-hoc tests: $ES_{40KO\text{-}WT}$=0.0361 [-0.2259, 0.2981], P-$value_{40KO\text{-}WT}$=0.9482; $ES_{41KO\text{-}WT}$=-0.0817 [-0.3438, 0.1802], P-$value_{41KO\text{-}WT}$=0.6690; $ES_{DKO\text{-}WT}$=-0.1496 [-0.2470, 0.2769], P-$value_{DKO\text{-}WT}$=0.9957.

IL13: ANOVA F(3,6)=4.534, P-value=0.0550, followed by Dunnett's post-hoc tests: $ES_{40KO\text{-}WT}$=-0.2847 [-0.5838, -0.00143], P-$value_{40KO\text{-}WT}$=0.0501; $ES_{41KO\text{-}WT}$=-0.2756 [-

bioRxiv preprint doi: https://doi.org/10.1101/2023.02.13.528372; this version posted February 13, 2023. The copyright holder for this preprint (which was not certified by peer review) is the author/funder, who has granted bioRxiv a license to display the preprint in perpetuity. It is made available under aCC-BY 4.0 International license.

0.5747, -0.0023], P-value$_{41KO-WT}$=0.0500; ES$_{DKO-WT}$=-0.3076 [-0.6067, -0.0085], P-value$_{DKO-WT}$=0.0448.

IL16: ANOVA F(3,6)=2.354, P-value=0.1712, followed by Dunnett's post-hoc tests: ES$_{40KO-WT}$=-0.2548 [-0.5799, 0.0701], P-value$_{40KO-WT}$=0.1168; ES$_{41KO-WT}$=-0.2251 [-0.5501, 0.0999], P-value$_{41KO-WT}$=0.1685; ES$_{DKO-WT}$=-0.1566 [-0.4817, 0.1684], P-value$_{DKO-WT}$=0.3817.

IL8: ANOVA F(3,3)=2.410, P-value=0.2445, followed by Dunnett's post-hoc tests: ES$_{40KO-WT}$=-0.2953 [-0.7676, 0.1770], P-value$_{40KO-WT}$=0.1553; ES$_{41KO-WT}$=-0.1517 [-0.6240, 0.3206], P-value$_{41KO-WT}$=0.4876; ES$_{DKO-WT}$=-0.1172 [-0.5895, 0.3550], P-value$_{DKO-WT}$=0.6382.

MIF: ANOVA F(3,6)=2.065, P-value=0.2064, followed by Dunnett's post-hoc tests: ES$_{40KO-WT}$=-0.2606 [-0.7211, 0.1999], P-value$_{40KO-WT}$=0.2776; ES$_{41KO-WT}$=-0.3457 [-0.8062, -0.0011], P-value$_{41KO-WT}$=0.0491; ES$_{DKO-WT}$=-0.1342 [-0.5947, 0.3263], P-value$_{DKO-WT}$=0.7083.

SERPINE1: ANOVA F(3,6)=1.079, P-value=0.4264, followed by Dunnett's post-hoc tests: ES$_{40KO-WT}$=-0.2159 [-0.6675, 0.2357], P-value$_{40KO-WT}$=0.3872; ES$_{41KO-WT}$=-0.2367 [-0.6883, 0.2150], P-value$_{41KO-WT}$=0.3263; ES$_{DKO-WT}$=-0.1515 [-0.6031, 0.3001], P-value$_{DKO-WT}$=0.6255.

CCL2: ANOVA F(3,6)=6.951, P-value=0.0223, followed by Dunnett's post-hoc tests: ES$_{40KO-WT}$=-0.2371 [-0.4208, -0.0533], P-value$_{40KO-WT}$=0.0173; ES$_{41KO-WT}$=-0.2109 [-0.3946, -0.0271], P-value$_{41KO-WT}$=0.0288; ES$_{DKO-WT}$=-0.0887 [-0.2714, 0.0961], P-value$_{DKO-WT}$=0.3887.

IL18: ANOVA F(3,6)=2.551, P-value=0.1129, followed by Dunnett's post-hoc tests: ES$_{40KO-WT}$=-0.2271 [-0.4208, 0.0633], P-value$_{40KO-WT}$=0.1067; ES$_{41KO-WT}$=-0.1909 [-0.3946, 0.0471], P-value$_{41KO-WT}$=0.1175; ES$_{DKO-WT}$=-0.1887 [-0.2714, 0.00961], P-value$_{DKO-WT}$=0.0763.

IL1RA: ANOVA F(3,6)=2.032, P-value=0.2109, followed by Dunnett's post-hoc tests: ES$_{40KO-WT}$=-0.1429 [-0.4931, 0.2073], P-value$_{40KO-WT}$=0.4957; ES$_{41KO-WT}$=-0.1878 [-0.5380, 0.1624], P-value$_{41KO-WT}$=0.3114; ES$_{DKO-WT}$=-0.2722 [-0.6224, -0.0077], P-value$_{DKO-WT}$=0.0493.

bioRxiv preprint doi: https://doi.org/10.1101/2023.02.13.528372; this version posted February 13, 2023. The copyright holder for this preprint (which was not certified by peer review) is the author/funder, who has granted bioRxiv a license to display the preprint in perpetuity. It is made available under aCC-BY 4.0 International license.

C. Levels of cytokines secreted in conditioned media quantified as target density (i.e. mean target spot intensity multiplied by target spot area) normalized to reference density (i.e. mean reference spot intensity multiplied by reference spot area). log2(fold change) is calculated with SCR MACs as reference. Differences of means between groups were tested using one-way ANOVA with repeated measures followed by Dunnett's post-hoc test. N=3/group

MIP1α: ANOVA F(3,6)=5.935, P-value=0.0315, followed by Dunnett's post-hoc tests: $ES_{40KD\text{-}SCR}$=-0.2016 [-0.3663, -0.0368], P-$value_{40KD\text{-}SCR}$=0.0219; $ES_{41KD\text{-}SCR}$=-0.1603 [-0.3250, 0.0044], P-$value_{41KD\text{-}SCR}$=0.0555; $ES_{DKD\text{-}SCR}$=-0.0632 [-0.2279, 0.1015], P-$value_{DKD\text{-}SCR}$=0.5377.

MIF: ANOVA F(3,6)=6.201, P-value=0.0287, followed by Dunnett's post-hoc tests: $ES_{40KD\text{-}SCR}$=-0.2372 [-0.4769, 0.00257], P-$value_{40KD\text{-}SCR}$=0.0521; $ES_{41KD\text{-}SCR}$=-0.2244 [-0.4641, 0.0152], P-$value_{41KD\text{-}SCR}$=0.0640; $ES_{DKD\text{-}SCR}$=-0.3173 [-0.5570, -0.0773], P-$value_{DKD\text{-}SCR}$=0.0154.

IL8: ANOVA F(3,6)=2.734, P-value=0.1361, followed by Dunnett's post-hoc tests: $ES_{40KD\text{-}SCR}$=-0.1798 [-0.3958, 0.0363], P-$value_{40KD\text{-}SCR}$=0.0965; $ES_{41KD\text{-}SCR}$=-0.0678 [-0.2840, 0.1482], P-$value_{41KD\text{-}SCR}$=0.6653; $ES_{DKD\text{-}SCR}$=-0.1504 [-0.3665, 0.0655], P-$value_{DKD\text{-}SCR}$=0.1661.

IL1RA: ANOVA F(3,6)=32.74, P-value=0.0004, followed by Dunnett's post-hoc tests: $ES_{40KD\text{-}SCR}$=-0.5182 [-0.7117, -0.3246], P-$value_{40KD\text{-}SCR}$=0.0004; $ES_{41KD\text{-}SCR}$=-0.5519 [-0.7454, -0.3583], P-$value_{41KD\text{-}SCR}$=0.0003; $ES_{DKD\text{-}SCR}$=-0.3719 [-0.5655, -0.1783], P-$value_{DKD\text{-}SCR}$=0.0025.

IL1β: ANOVA F(3,6)=2.989, P-value=0.1177, followed by Dunnett's post-hoc tests: $ES_{40KD\text{-}SCR}$=-0.2059 [-0.9746, 0.5628], P-$value_{40KD\text{-}SCR}$=0.7523; $ES_{41KD\text{-}SCR}$=-0.1785 [-0.9472, 0.5902], P-$value_{41KD\text{-}SCR}$=0.8159; $ES_{DKD\text{-}SCR}$=-0.7064 [-1.475, 0.0623], P-$value_{DKD\text{-}SCR}$=0.0684.

ICAM1: ANOVA F(3,6)=14.42 P-value=0.0038, followed by Dunnett's post-hoc tests: $ES_{40KD\text{-}SCR}$=-0.1981 [-0.2943, -0.1019], P-$value_{40KD\text{-}SCR}$=0.0017; $ES_{41KD\text{-}SCR}$=-0.1339 [-0.2301, -0.0375], P-$value_{41KD\text{-}SCR}$=0.0122; $ES_{DKD\text{-}SCR}$=-0.1339 [-0.2301, -0.0377], P-$value_{DKD\text{-}SCR}$=0.0122.

bioRxiv preprint doi: https://doi.org/10.1101/2023.02.13.528372; this version posted February 13, 2023. The copyright holder for this preprint (which was not certified by peer review) is the author/funder, who has granted bioRxiv a license to display the preprint in perpetuity. It is made available under aCC-BY 4.0 International license.

CXCL11: ANOVA $F(3,6)=4.333$ P-value=0.0601, followed by Dunnett's post-hoc tests: $ES_{40KD\text{-}SCR}$=-0.1663 [-0.3380, 0.0054], $P\text{-value}_{40KD\text{-}SCR}$=0.0565; $ES_{41KD\text{-}SCR}$=-0.1148 [-0.2865, 0.0568], $P\text{-value}_{41KD\text{-}SCR}$=0.1856; $ES_{DKD\text{-}SCR}$=-0.1790 [-0.3508, -0.0072], $P\text{-value}_{DKD\text{-}SCR}$=0.0425.

CCL5: ANOVA $F(3,6)=15.64$ P-value=0.0031, followed by Dunnett's post-hoc tests: $ES_{40KD\text{-}SCR}$=-0.1876 [-0.2799, -0.0953], $P\text{-value}_{40KD\text{-}SCR}$=0.0018; $ES_{41KD\text{-}SCR}$=-0.1615 [-0.2537, -0.0692], $P\text{-value}_{41KD\text{-}SCR}$=0.0040; $ES_{DKD\text{-}SCR}$=-0.1293 [-0.2216, -0.0371], $P\text{-value}_{DKD\text{-}SCR}$=0.0119.

TNFα: ANOVA $F(3,6)=93.00$ P-value<0.0001, followed by Dunnett's post-hoc tests: $ES_{40KD\text{-}SCR}$=-0.6826 [-0.9495, -0.4157], $P\text{-value}_{40KD\text{-}SCR}$=0.0005; $ES_{41KD\text{-}SCR}$=-0.7145 [-0.9814, -0.4476], $P\text{-value}_{41KD\text{-}SCR}$=0.0004; $ES_{DKD\text{-}SCR}$=-1.438 [-1.705, -1.171], $P\text{-value}_{DKD\text{-}SCR}$<0.0001.

SERPINE1: ANOVA $F(3,6)=8.744$ P-value=0.0131, followed by Dunnett's post-hoc tests: $ES_{40KD\text{-}SCR}$=-0.2327 [-0.3997, -0.0657], $P\text{-value}_{40KD\text{-}SCR}$=0.0122; $ES_{41KD\text{-}SCR}$=-0.0641 [-0.2312, 0.1028], $P\text{-value}_{41KD\text{-}SCR}$=0.5366; $ES_{DKD\text{-}SCR}$=-0.2097 [-0.3766, -0.0427], $P\text{-value}_{DKD\text{-}SCR}$=0.0196.

Effect sizes (ES) are reported as unstandardized point estimates with 95% confidence intervals in the same unit as depicted on each graph

Supplementary Table legends

Supplementary Table 1

LAM_datasets sheet. Human and mouse lipid-associated macrophage (LAM) sc/snRNA-seq datasets used to reconstruct gene regulatory networks.

LAM_genesets sheet. Human and mouse LAM genesets used in regulon enrichment, RRHO, and other analyses (genes listed in alphabetical order).

LAM_TFs sheet. Candidate human and mouse LAM TFs nominated in more than half human and mouse GRNs, respectively (listed in alphabetical order).

Supplementary Table 2

bioRxiv preprint doi: https://doi.org/10.1101/2023.02.13.528372; this version posted February 13, 2023. The copyright holder for this preprint (which was not certified by peer review) is the author/funder, who has granted bioRxiv a license to display the preprint in perpetuity. It is made available under aCC-BY 4.0 International license.

BHLHE40/41_regulons sheet. BHLHE40 and BHLHE41 human and mouse regulons from meta-analysed human and mouse GRNs. Meta-analyzed GRNs were generated by aggregating all the bootstraps from individual networks generated by ARACNe.

LAM_genes_in_BHLHE40/41_regulons sheet. Human and mouse LAM genes in BHLHE40 and BHLHE41 human and mouse regulons (see BHLHE40/41_regulons sheet). Human LAM genes in BHLHE40 and BHLHE41 human regulons. LAM genes were selected from Jaitin *et al*. [3] (Dataset S6, FDR Adj.P-value < 0.05). Mouse LAM genes in Bhlhe40 and Bhlhe41 mouse regulons. LAM genes were selected from Keren-Shaul *et al*. [10] (Table S3, FDR Adj.P-value < 0.05).

LAM_genes_with_promoter_proxy-bound_by_BHLHE40/41 sheet. Human lipid-associated macrophage (LAM) genes with promoters proxy-bound by BHLHE40 and/or BHLHE41. LAM genes were selected from Jaitin *et al*. [3] (Dataset S6, FDR Adj.P-value < 0.05).

Genes_in_most_significant-RRHO_overlaps sheet. Genes in most significant overlaps from rank-rank hypergeometric overlap (RRHO) analyses. Z scores (Z.std) from RNA-seq differential gene expression analyses were assigned to each overlapping gene to run Ingenuity Pathway Analysis (IPA). Human LAM signature genes in Figure 4A and Supplementary Figure 7A were selected from Jaitin *et al.* [3] (Dataset S6, FDR Adj.P-value < 0.05). Mouse LAM signature genes in Figure 7A were selected from Keren-Shaul *et al.* [10] (Table S3, FDR Adj.P-value < 0.05).

Supplementary Table 3. Differential gene expression (DGEA) and gene set enrichment (GSEA) analysis of *BHLHE40* and/or *BHLHE41* knockout (KO) in human iPSC-derived microglia (iMGLs). 40KO = BHLHE40 KO iMGLs, 41KO = BHLHE41 KO iMGLs, DKO = BHLHE40 and BHLHE41 double KO iMGLs, WT = iMGLs derived from the parental iPSC line.

Supplementary Table 4. Differential gene expression (DGEA) and gene set enrichment (GSEA) analysis of *BHLHE40* and/or *BHLHE41* knockdown (KD) in human THP-1 macrophages (MACs). 40KD = MACs treated with BHLHE40 siRNA,

bioRxiv preprint doi: https://doi.org/10.1101/2023.02.13.528372; this version posted February 13, 2023. The copyright holder for this preprint (which was not certified by peer review) is the author/funder, who has granted bioRxiv a license to display the preprint in perpetuity. It is made available under aCC-BY 4.0 International license.

41KD = MACs treated with BHLHE41 siRNA, DKD = MACs treated with BHLHE40 and BHLHE41 siRNA, SCR = MACs treated with scrambled siRNA.

Supplementary Table 5. Differential gene expression (DGEA) and gene set enrichment (GSEA) analysis of *Bhlhe40* and *Bhlhe41* double knockout (DKO) in mouse microglia. DKO = Bhlhe40/41 DKO mouse microglia, compared to microglia derived from wild-type control mice.

Supplementary Table 6. Sequences of primers used for RT-qPCR and sequences of guide RNAs, single-stranded oligodeoxynucleotides (ssODNs), and PCR primers used for CRISPR/Cas9-mediated HDR.
